# Time-Dependent DCE-MRI Radiomics to Predict Response to Neoadjuvant Therapy in Breast Cancer: A Multicenter Study with External Validation

**DOI:** 10.3390/diagnostics16040611

**Published:** 2026-02-19

**Authors:** Giulia Vatteroni, Riccardo Levi, Paola Nardi, Giulia Pruneddu, Elisa Salpietro, Federica Fici, Cinzia Monti, Rubina Manuela Trimboli, Daniela Bernardi

**Affiliations:** 1 Department of Biomedical Sciences, Humanitas University, Via R. Montalcini 4, 20072 Pieve Emanuele, Italydaniela.bernardi@hunimed.eu (D.B.); 2 Radiology Department, IRCCS Humanitas Research Hospital, Via A. Manzoni 56, 20089 Rozzano, Italy; 3 Radiology Department, Humanitas Gavazzeni, Via Mauro Gavazzeni 21, 24125 Bergamo, Italy

**Keywords:** neo-adjuvant therapy, breast cancer, MRI, radiomics, machine learning, response prediction

## Abstract

**Background**: The accurate prediction of response to neoadjuvant therapy (NAT) is crucial for optimizing breast cancer management. Conventional breast Dynamic Contrast-Enhanced Magnetic Resonance Imaging (DCE-MRI) radiomics typically relies on single post-contrast phases and may not fully capture temporal enhancement patterns related to tumor heterogeneity. This study evaluated a machine learning model based on time-dependent radiomic features extracted from pre-treatment DCE-MRI for predicting NAT response in breast cancer patients. **Methods**: Breast DCE-MRI examinations of women scheduled for NAT, acquired on 1.5 T scanners from three different vendors, were retrospectively collected from two centers. Tumors were automatically segmented on the third post-contrast DCE image using a 3D nnUNet model trained on 30 lesions. All DCE phases were registered to the reference image, and radiomic features were extracted from a consistent tumor region of interest across all phases. Time-dependent radiomic features were computed using linear regression modeling of feature evolution over time. A random forest classifier integrating static and time-dependent radiomic features was developed to predict pathological complete response (pCR), partial response (pPR), and non-response (pNR). Model performance was evaluated using internal validation (Center 1) and an independent external test cohort (Center 2). **Results**: A total of 212 patients were included (173 from Center 1 and 39 from Center 2), comprising 103 pCR, 103 pPR and 6 pNR cases. Among 759 extracted features, 30 showed significant differences across response groups. Several time-dependent texture features related to intratumoral heterogeneity were significantly associated with pNR. The model achieved AUC values of 0.80, 0.81, and 0.95 in the internal validation cohort and 0.75, 0.74, and 0.86 in the external test cohort for predicting pCR, pPR, and pNR, respectively. **Conclusions**: Time-dependent radiomic features derived from pre-treatment breast DCE-MRI enable the accurate prediction of response to NAT, with particularly strong performance in identifying non-responders. This approach may support imaging-based risk stratification and contribute to more personalized treatment.

## 1. Background

Neoadjuvant therapy (NAT) has become an integral part of breast cancer management [1]. Initially used for locally advanced or inoperable tumors to enable breast-conserving surgery, NAT is now routinely administered to patients with lymph node-positive disease and to those with biologically aggressive early-stage tumors, including high-grade, HER2-positive, and hormone receptor-negative subtypes [1,2,3,4].

Several clinical trials have demonstrated that achieving a pathological complete response (pCR) following NAT is associated with improved disease-free survival and overall survival [5,6,7,8]. However, despite substantial response rates—reaching up to 55% in hormone receptor-positive/HER2-positive tumors and up to 90% in hormone receptor-negative/HER2-positive tumors [9,10]—a large proportion of patients fail to achieve pCR [11,12].

The accurate prediction of response to NAT is essential to optimize treatment strategies, as it may allow early risk stratification, reduce unnecessary toxicity and support timely treatment adaptation. In particular, the early identification of non-responders remains a major unmet clinical need, as these patients derive limited benefit from standard NAT regimens and may require alternative therapeutic approaches [11,12,13].

International guidelines recommend breast Dynamic Contrast-Enhanced Magnetic Resonance Imaging (DCE-MRI) before and after NAT to assess treatment response, owing to its high sensitivity and ability to capture tumor vascularity and functional characteristics [14,15].

In clinical practice, response to NAT is mainly evaluated through visual assessments of tumor size, volume, and enhancement patterns on MRI, with categorization according to the RECIST v1.1 criteria [16]. In selected cases, diffusion-weighted imaging (DWI) and quantitative apparent diffusion coefficient (ADC) measurements have been shown to improve response assessment [17,18,19]. However, these approaches are limited by subjectivity, inter-reader variability, and an incomplete characterization of tumor biology.

Despite the widespread use of imaging for response assessment during NAT, its translation into response-guided treatment remains limited. A recent systematic review by Lafci et al. [20] showed that, although imaging is employed in more than 80% of NAT breast cancer trials, treatment modification based on imaging response occurs in only a small fraction of cases, largely due to the lack of standardized imaging protocols and actionable response criteria.

In recent years, MRI-based radiomics has emerged as a promising imaging biomarker for predicting treatment response by extracting quantitative features that reflect tumor heterogeneity and microstructural organization [13,21,22,23,24]. Several studies have demonstrated the potential of radiomics and machine learning (ML) approaches applied to pre-treatment MRI to predict pathological complete response [13,25,26,27,28,29]. Recent evidence further suggests that texture-based radiomic descriptors of intratumoral heterogeneity may provide complementary prognostic information when integrated with conventional radiological assessment, improving patient stratification beyond radiological or radiomic features alone [30].

However, most existing models rely on static radiomic features extracted from a single post-contrast phase, potentially overlooking clinically relevant temporal information embedded in DCE-MRI [31].

DCE-MRI inherently provides time-resolved information on contrast uptake and washout, reflecting tumor perfusion, vascular permeability, and microenvironmental heterogeneity [4,14,32,33,34,35]. These dynamic properties are closely related to treatment sensitivity and resistance, suggesting that time-dependent radiomic imaging biomarkers may offer additional predictive value beyond static features. Nevertheless, the integration of temporal radiomics into ML-based prediction models remains limited, and external validation across independent institutions is often lacking, raising concerns about generalizability and clinical translation [12,32].

Together, these findings highlight the need for integrated, quantitative and reproducible imaging approaches capable of translating imaging information into clinically meaningful patient stratification.

To address these limitations, we conducted a retrospective multicenter study including breast cancer patients undergoing NAT at two independent institutions. Using pre-treatment DCE-MRI, we developed and validated a ML-based prediction model integrating static and time-dependent radiomic features to stratify patients according to pCR, pathological partial response (pPR) and pathological non-response (pNR). Data from one center were used for model development and internal validation, while data from the second center served as an independent external test cohort enabling an objective assessment of model robustness and generalizability.

## 2. Methods

### 2.1. Study Design and Population

This retrospective study was approved by the institutional review board (protocol number: 59/24) and conducted at two centers in Milan (Center 1) and in Bergamo (Center 2). Study procedures adhered to the CLEAR (CheckList for EvaluAtion of Radiomics) reporting guidelines (Supplementary Material S1) [36]. The requirement for informed consent was waived due to the retrospective nature of the study. All patient data were handled in compliance with the principles of good scientific practice.

Consecutive women with newly diagnosed breast cancer who were candidates for NAT and underwent breast DCE-MRI at Humanitas Research Hospital or Humanitas Gavazzeni between January 2019 to May 2023 were retrospectively evaluated.

Women were included if pre- and post-treatment MRI and surgical pathology results were available. Exclusion criteria included incomplete examinations or poor image quality that precluded adequate lesion segmentation.

NAT regimens were administered according to standard protocol outlined in the National Comprehensive Cancer Network (NCCN) guidelines for breast cancer [15] based on the histological subtype. Before starting NAT, all lesions were marked with a non-magnetic clip to enable the localization of the original tumor site, particularly in patients achieving pCR.

For each patient, the following variables were recorded: age, histologic subtype, tumor grade, receptor status, proliferation rate (Ki67), tumor size on pre- and post-NAT DCE-MRI, T and N stage on pre-and post-NAT DCE-MRI, and NAT regimens.

### 2.2. Magnetic Resonance Imaging Acquisition

Patients underwent breast DCE-MRI at two time points: before NAT (within two weeks prior to treatment initiation) and after NAT (within two weeks after the last treatment cycle).

All DCE-MRI examinations were performed with patients in the prone position using 1.5 T scanners from three different vendors: Magnetom Avanto (Siemens Healthcare, Erlangen, Germany), Signa HDx (GE Healthcare, Little Chalfont, UK), and Achieva Tx (Philips Medical Systems, Best, the Netherlands). Scanners were equipped with dedicated bilateral breast coils (16-channel for Siemens; 8-channel for GE and Philips systems).

For each patient, a standardized multiparametric protocol was employed, including an axial T2-weighted STIR sequence, an axial DWI echo-planar sequence (b values of 50 and 800 s/mm^2^), and an axial T1-weighted 3D gradient-echo sequence. The T1-weighted dynamic sequence was acquired once before and six times after intravenous administration of 0.1 mmol/kg of Gadobutrol (Gadovist^®^, Bayer, Berlin, Germany) using a power injector at a flow rate of 2 mL/s. Post-processed subtraction images, multiplanar reconstruction (MPRs), and maximum intensity projection (MIP) images were subsequently generated.

### 2.3. Molecular Subtype Classification

Hormone receptor (HR) status was assessed by immunohistochemistry (IHC), considering estrogen receptor (ER) and progesterone receptor (PR) positivity when immunoreactivity was present in more than 1% of tumor cells. HER2 status was also evaluated by IHC, using a standardized scoring system ranging from 0 to 3+. Tumors scoring 0 or 1+ were classified as HER2-negative, whereas tumors scoring 3+ or showing a 2+ score with confirmed gene amplification on fluorescence in situ hybridization (FISH) were classified as HER2-positive (HER2+). Patients with ER and/or PR positivity and HER2-negative status were categorized as HR-positive, while those with HER2 overexpression were categorized as HER2-positive, regardless of hormone receptor expression. Luminal B HER2+ cancers were included in the HER2-positive group based on the biological relevance of HER2 overexpression, which strongly influences prognosis and therapeutic strategy.

Patients who were negative for both HR and HER2 were classified as in triple-negative. Therapeutic regimens were assigned according to the molecular subtype classification.

### 2.4. Reference Standard and Definition of Pathological Response

At the end of NAT, all patients underwent surgery, and the histopathological assessment of the surgical specimen served as the reference standard. Pathological response was evaluated by a breast pathologist with 15 years of experience using the Pinder classification [37,38] (Table 1).

For the purpose of this study, patients with Pinder 1i (absence of invasive cancer without residual ductal carcinoma in situ) and Pinder 1ii (absence of invasive cancer with the presence of ductal carcinoma in situ) were classified as pCR.

Patients with Pinder 2i, 2ii, or 2iii were classified as pPR and those with Pinder 3 were classified as pNR.

### 2.5. Image Analysis

#### 2.5.1. Tumor Segmentation

Subtracted images acquired at tumor peak enhancement (third DCE-T1-weighted sequence) from the pre-treatment DCE-MRI, approximately two minutes after contrast administration, were used for volumetric segmentation, as tumors are best visualized at this time point.

**Figure 1:**
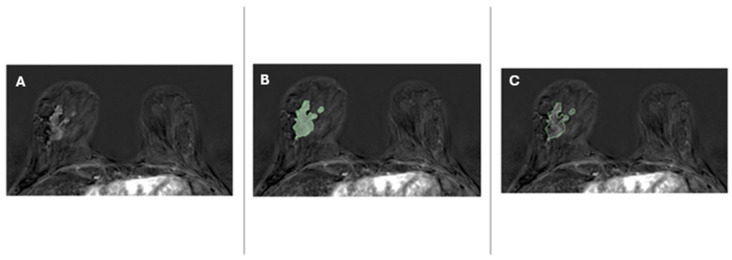
A 52-year-old woman with invasive ductal carcinoma (G2, luminal B HER2+) of the right breast. The image shows an example of segmented malignant breast lesion candidate for NAT. To enhance the graphical representation, a cropped version of a 512 × 512 pixel subtracted dynamic contrast-enhanced MRI (DCE-MRI) is displayed. All images maintain the same zoom level. The MRI reveals a mass measuring 45 mm in diameter. The lesion exhibits strong post-contrast enhancement with a high wash-in rate and a type II enhancement curve. (**A**) Original axial post-contrast fat-saturated T1-weighted subtracted image from post-NAT breast MRI; (**B**) overlaid segmentation on the original image, highlighting the lesion; (**C**) contour of the segmented region on the original image, outlining the lesion’s borders.

All histologically confirmed invasive tumors were manually segmented using open-source research software (3D Slicer, version 4.10.2) (Figure 1). Radiology reports were available during segmentation, and image windowing was adjusted to optimize lesion visualization. Axillary lymph nodes were considered a non-measurable disease and were excluded from the segmentation.

A convolutional neural network (CNN) based on the nnU-Net framework was trained on 30 manually segmented cases (training set) by a radiology resident with 2 years of experience in breast imaging, under the supervision of a senior breast radiologist with more than 10 years of experience. Model training parameters are reported in Supplementary Material S2. After training, the CNN was applied to automatically segment the entire dataset.

Segmentation accuracy was evaluated using the DICE similarity coefficient (DSC) between manual and automated segmentations. In the external cohort, all DCE-MRI images were segmented by a single radiologist and compared with the CNN-generated volumes. The mean DSC achieved was 0.90 (range: 0.80–0.99) in the internal cohort and 0.94 (range: 0.61–0.99) in the external cohort, indicating an excellent agreement between manual and automated segmentation.

#### 2.5.2. Radiomic Feature Extraction

Radiomic features were computed from all six sequences of DCE-T1-weighted sequences. The tumor segmentation obtained from the CNN model was superimposed onto all the DCE-T1w sequences using an affine registration algorithm (SimpleITK library). Each DCE-MRI volume was normalized for signal intensity using N4 bias-field correction (SimpleITK library). Radiomic features were then extracted from each volume of interest (VOI) with the *Pyradiomics* library. Complete extraction parameters and settings are reported in Supplementary Material S3.

To characterize the temporal evolution of radiomic features across the dynamic phases, a linear regression model was fitted to each feature for every patient using the least-squares method (SciPy *linregress* function, default settings without regularization or weighting). The order of DCE acquisitions was treated as the time variable (independent variable), and the corresponding feature values across phases served as the dependent variable. This modeling approach yielded two parameters for each feature: the slope, representing the rate of change in the feature over time, and the intercept, representing its estimated baseline value before contrast enhancement.

These parameters were considered time-dependent features and used as independent predictors for model training. For each original radiomic feature, two derived variables (slope and intercept) were generated. No further feature combination or dimensionality reduction was applied.

### 2.6. Machine Learning Model

A ML model was developed to predict pCR, pPR and pNR. The dataset from Center 1 was randomly divided into a training set (75%) and a test set (25%), while the dataset from Center 2 served as an external validation cohort.

In addition to the primary analysis, an exploratory subgroup analysis was performed by further stratifying patients according to refined Pinder categories (Pinder 1i, 1ii, and ≥2) to investigate whether time-dependent radiomic features differed between true complete responders and cases with residual in situ or invasive disease. Detailed methods are reported in Supplementary Material S4.

Radiomic features were standardized using z-score normalization, computed from the Center 1 training set and applied to both the Center 1 test and Center 2 validation set. Feature selection was performed using recursive feature elimination (RFE) with 5-fold cross-validation, optimizing the area under the receiver operating characteristic curve (AUC) in a one-vs-rest configuration.

The most discriminative features identified by RFE were used to train a random forest classifier. Model hyperparameters were optimized via nested cross-validation using a Bayesian Tree-Parzen Estimator (B-TPE) implemented in Optuna (v4.4.0). Model performance was then evaluated on the test sets in terms of accuracy, sensitivity, specificity, and macro-averaged AUC.

Additionally, the final ML model was compared with a reference model trained using clinical variables only, including parameters and tumor extent on DCE-MRI. Eventually, the prediction of both radiomics-based ML model and clinical-based ML were combined together using late-fusion strategy to assess the combined effect on target prediction.

### 2.7. Statistical Analysis

Continuous variables were assessed for normality using the Shapiro–Wilk test. Differences among the three pathological response groups (pCR, pPR, pNR) were evaluated using one-way analysis of variance (ANOVA) for normally distributed variables and the Kruskal–Wallis test for non-normally distributed variables.

For the evaluation of the ML models, classification performance was quantified using accuracy, sensitivity, specificity, and macro-averaged area under the receiver operating characteristic curve (AUC). Receiver operating characteristic (ROC) curves were generated using a one-vs-rest approach to account for the multiclass nature of the classification problem.

Comparisons of model performance, including AUC values, were performed using the DeLong test. All statistical tests were two-sided, and statistical significance was defined as a *p*-value of < 0.05. Statistical analyses and machine learning experiments were performed in Python (version 3.9) using the SciPy and scikit-learn libraries.

All analyses were conducted in accordance with the current recommendations for statistical reporting in radiomics research.

## 3. Results

### 3.1. Patients

During the study period, 242 women candidates for NAT who underwent breast MRI at two hospitals—Center 1 (Humanitas Research Hospital) and Center 2 (Humanitas Gavazzeni)—were identified. A total of 173 patients from Center 1 and 39 patients from Center 2 were included in the analysis. Thirty patients were excluded: 23 due to poor image quality (15 related to motion artifacts, 5 to susceptibility artifacts, and 3 to inadequate fat suppression) and 7 due to missing post-treatment examination.

For Center 1, the median age was 51 years [interquartile range (IQR): 46–59] and the median tumor size before NAT was 3.0 cm (IQR: 2.2–4.5 cm). Among the 173 patients, 83 (48%) achieved pCR, 86 (50%) were classified as pPR, and 4 (2%) as pNR. Molecular subtype distribution was 16 HR-positive, 99 HER2-positive, and 58 triple-negative.

For Center 2, the median age was 50 years (IQR: 43–56), and the median tumor size before NAT was 4.6 cm (IQR: 2.7–5.5 cm). Among the 39 patients, 20 (51%) achieved pCR, 17 (44%) were classified as pPR, and 2 (5%) as pNR.

Molecular subtype distribution was 12 HR-positive, 16 HER2-positive, and 11 triple-negative. Type of response according to molecular histotypes is summarized in Supplementary Material S5.

Patients and tumor characteristics of both centers are summarized in Table 2.

HR-positive/HER2-negative patients primarily received anthracycline- and/or taxane-based chemotherapy [e.g., AC (Doxorubicin + Cyclophosphamide) ± taxane]. HER2-positive patients were treated with chemotherapy combined with HER2-targeted agents, including trastuzumab alone or in combination with pertuzumab. Regimens included AC + taxane + trastuzumab, taxane + trastuzumab ± pertuzumab, and, in a minority of cases, carboplatin-based combinations.

Triple-negative breast cancer patients received anthracycline- and taxane-based chemotherapy, with carboplatin added in selected cases. A subgroup also received immunotherapy with pembrolizumab combined with chemotherapy.

Overall, 14 of 242 patients (5.8%) discontinued therapy prematurely (11 in Center 1 and 3 in Center 2) due to adverse events such as hypersensitivity reactions, neutropenia, or atrial fibrillation.

Treatment regimens are summarized in Supplementary Material S6.

### 3.2. Radiomic Feature Analysis

A total of 759 radiomic features were extracted and analyzed from the tumor volumes of interest (VOIs) for each patient. After computing the slope and intercept for each feature across the DCE-MRI sequences, the total number of derived features increased to 3920.

**Figure 2:**
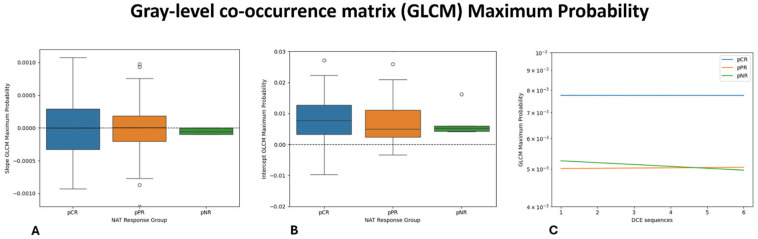
Boxplot and Lineplot showing the dynamic changes in the GLCM Maximum Probability: (**A**): slope; (**B**): intercept; and (**C**): average linear regression model of each NAT response group.

**Figure 3:**
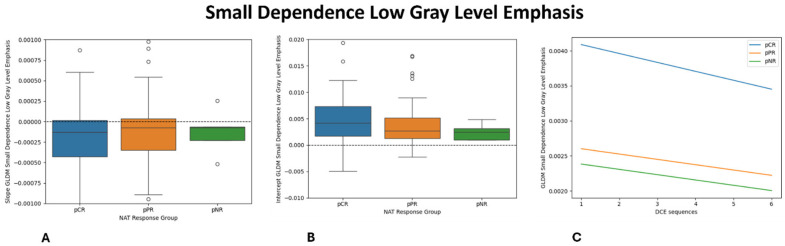
Boxplot and Lineplot showing the dynamic changes in the GLDM Small-Dependence Low-Gray-Level Emphasis: (**A**): slope (**B**): intercept (**C**): and average linear regression model of each NAT response group.

Univariate analysis identified 30 features that differ significantly among the three response groups (pCR, pPR, and pNR). Among these, the gray-level co-occurrence matrix (GLCM) Maximum Probability (*p* = 0.009) and the intercept of Small-Dependence Low-Gray-Level Emphasis (SDLGLE) (*p* = 0.010) showed the strongest associations with pNR. The distribution of these features across response groups are shown in Figure 2 and Figure 3.

Regarding feature selection for the ML model, the recursive feature elimination with cross-validation (RFECV) procedure selected eight features, which were subsequently used for model training.

### 3.3. Machine Learning Model Performance

The final radiomics models achieved an overall accuracy of 67.4% (31/42), a macro-averaged sensitivity of 56.5%, and a specificity of 78.9% in the internal validation cohort.

In the external validation cohort, the model reached an accuracy of 72.5% (28/39), a macro-averaged sensitivity of 65.6%, and a specificity of 83.1%.

**Figure 4:**
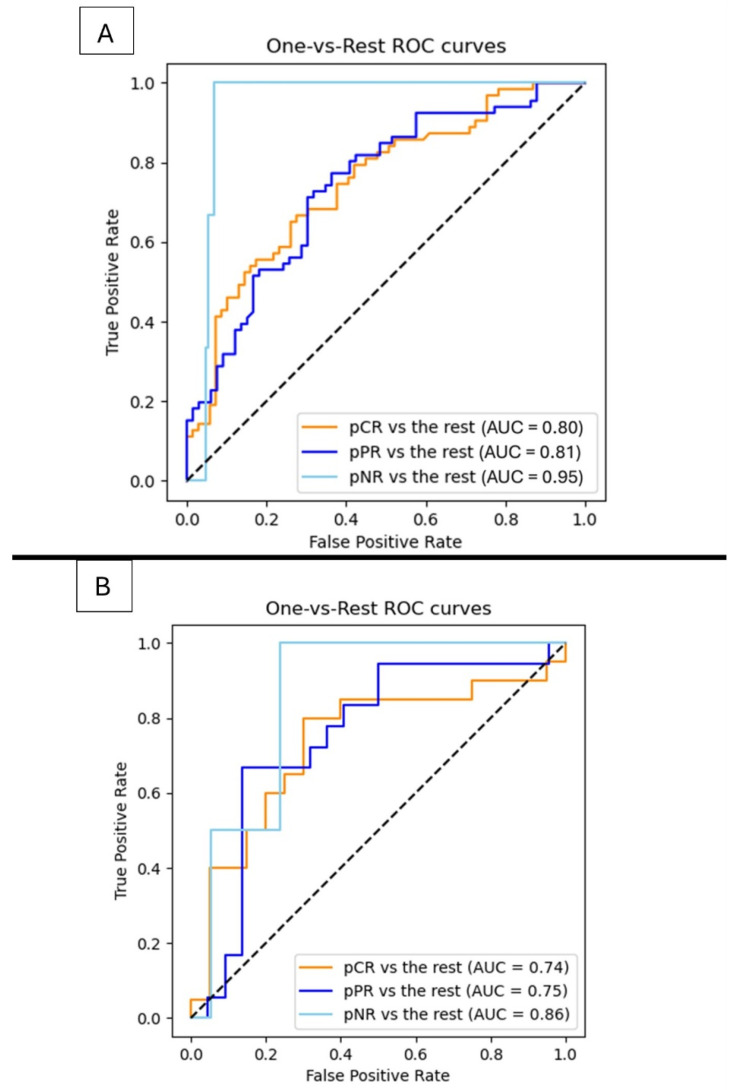
AUROC (one vs. rest) evaluated in Center 1 ((**A**); internal validation) and Center 2 ((**B**); external validation).

Regarding the ROC analysis, the model achieved an AUC of 0.80 for pCR vs. the rest, 0.81 for pPR vs. the rest, and 0.95 for pNR vs. the rest in the internal validation cohort (Figure 4). The corresponding AUCs in the external cohort were 0.74, 0.75, and 0.86, respectively.

Confusion matrices for both cohorts are provided in Supplementary Material S7.

**Figure 5:**
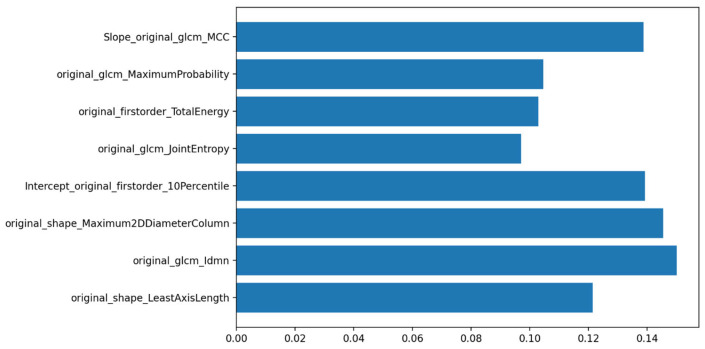
Features importance in prediction of pCR class, pPR class and pNR class.

Feature importance analysis identified GLCM Maximum Probability and the intercept of Small-Dependence Low-Gray-Level Emphasis (SDLGLE) as the most discriminative variables (Figure 5), consistent with the univariate statistical results.

For comparison, a clinical-only model—including histopathological and MRI-based tumor extension parameters—achieved similar AUCs of 0.80, 0.81, and 0.95 in the internal validation cohort and 0.75, 0.74, and 0.86 in the external cohort.

The late-fusion ML achieved lower performances and similar AUCs of 0.78, 0.75, and 0.85 in the internal validation cohort and 0.72, 0.70, and 0.84 in the external cohort.

The exploratory subgroup analysis results are summarized in Supplementary Material S4.

## 4. Discussion

In this study, we developed and validated an ML model based on time-dependent radiomic features extracted from pre-treatment DCE-MRI to predict response to NAT in breast cancer patients. This model successfully predicted patients’ pathological responses to NAT, with the highest performance observed in non-responders, the subgroup for whom early treatment adaptation and avoidance of unnecessary toxicity are most clinically relevant. On the external validation cohort, although performance slightly declined, the model retained good discriminative ability, supporting the robustness and potential transferability of the model, underscoring the potential of generalizing radiomics models across institutions due to differences in MRI acquisition protocols, scanners, and patient populations.

Although pCR is a key therapeutic goal in breast cancer owing to its association with improved disease-free and overall survival and its potential role in surgical de-escalation, it is achieved in only a minority of patients [8,39].

Evidence from recent clinical trials, such as TRAIN-3 [35], has demonstrated that a subset of patients can achieve pCR after only a few treatment cycles and that MRI may facilitate the early identification of responders, supporting treatment de-escalation strategies. Conversely, patients with resistant disease require early risk stratification to allow the timely modification of ineffective therapies and to avoid unnecessary delays in definitive surgery. In this context, the ability to accurately predict non-response before treatment initiation could have substantial clinical impact.

Currently, the histopathological evaluation of the surgical specimen remains the reference standard for assessing response to NAT [40]. However, this approach is inherently invasive and provides outcome information only after treatment completion.

Although imaging is widely used during NAT, its role in guiding treatment adaptation remains limited. As reported by a recent systematic review [20], imaging-guided treatment modification is implemented in only a small fraction of trials, mainly due to the lack of standardized protocols and quantitative, actionable imaging biomarkers.

Several strategies have been explored to predict response to NAT. Clinicopathological factors—such as molecular subtype, tumor grade, Ki-67, and baseline tumor burden—are known to influence treatment sensitivity but offer only static and indirect information. Imaging-based approaches have largely relied on morphological assessments with conventional MRI, including RECIST-based changes in tumor size and volume, which often occur late in the treatment course [4]. Functional imaging techniques, including DWI, DCE-MRI pharmacokinetic parameters, and PET/CT metabolic response, have also been investigated, with variable results [41].

These limitations highlight the need for non-invasive imaging biomarkers capable of enabling earlier and more informative response assessments capable of predicting response earlier and enabling the real-time monitoring of therapeutic effects. In this setting, DCE-MRI is particularly well suited for this purpose, as it allows the longitudinal assessment of tumor vascularity and perfusion, supporting treatment personalization based on imaging-derived functional information. More recently, radiomics and ML-based methods applied to MRI, DWI, or PET imaging have shown promising performance for response prediction [25].

Radiomics further extends the value of DCE-MRI enabling the quantitative characterization of intratumoral heterogeneity, capturing information beyond conventional visual interpretation.

Several studies have reported promising results in predicting pCR using radiomic features extracted from pre-treatment DCE-MRI [21,25,28,30,42,43,44,45,46,47].

However, many of these investigations have focused on binary classification (pCR vs. non-pCR), which may oversimplify the biological spectrum of treatment response. In contrast, our study extends prior work by incorporating time-dependent radiomic features derived from all dynamic phases of pre-treatment DCE-MRI, enabling a multiclass stratification of treatment response and providing an externally validated framework for the early identification of non-responders. This refined stratification is clinically meaningful, as it better reflects real-world decision-making scenarios and supports tailored strategies such as treatment escalation, early switching, or de-escalation.

In the primary analysis, our cohort exhibited an overall pCR rate of 50%, which is higher than the 40% reported in the ACOSOG Z1071 trial [48]. This difference may be explained by the high prevalence of HER2+ and triple-negative subtypes in our population, both of which are known to achieve higher response rates to NAT compared with luminal tumors [28,42,49]. When compared with the prior literature, our model achieved performance comparable or superior to previously published ML-based radiomics models [25,28,42]. Moreover, our findings are consistent with those reported by Xiong et al. [42] and Zhuang et al. [45], who also demonstrated the potential of time-dependent radiomic features for response prediction.

Among the 759 extracted radiomic features, 30 showed significant differences across response groups. In particular, GLCM Maximum Probability and the intercept of the Small-Dependence Low-Gray-Level Emphasis were strongly associated with pNR, suggesting that resistant tumors exhibit a more disorganized internal architecture. GLCM Maximum Probability reflects the uniformity of gray-level distribution within the tumor with lower values indicating a greater intratumoral heterogeneity—a feature consistently associated with poor treatment response. Conversely, higher values denote more homogeneous tissue organization, which was more frequently observed in tumors achieving pCR. The Small-Dependence Low-Gray-Level Emphasis, which captures fine textural variations in low-intensity regions related to structural disorganization, reached statistical significance primarily through its intercept parameter, suggesting baseline differences across response groups rather than dynamic temporal changes.

Overall, patients achieving pCR exhibited lower heterogeneity-related feature values, consistent with a more homogeneous and treatment-sensitive tumor composition, whereas non-responders displayed a higher baseline heterogeneity and a disorganized internal architecture—features characteristic of resistant phenotypes. Partial responders demonstrated intermediate values, supporting the concept of a biological continuum of tumor response rather than a dichotomous outcome.

Our supplementary exploratory subgroup analysis based on refined pathological response categories further supported this continuum, showing that cases with residual in situ disease (pCRis) exhibited enhancement dynamics intermediate between a true complete response and a partial response or non-response. Although hypothesis-generating, these findings suggest that residual in situ disease may retain microstructural and vascular characteristics distinct from both complete eradication and persistent invasive disease, providing additional biological insights into response heterogeneity.

These observations align with previous reports [4,50], indicating that a true complete response is associated with the early normalization of enhancement patterns, flattening of time–intensity curves, and disappearance of delayed washout, whereas a partial response or non-response corresponds to persistent enhancement irregularities and complex signal behavior. The integration of dynamic textural markers reinforces the hypothesis that temporal radiomics can detect subtle yet biologically meaningful differences in microvascular remodeling and tissue composition induced by therapy, contributing to a more physiologically grounded interpretation of treatment response. In such a perspective, tumor heterogeneity provides a biological explanation for the variable therapeutic responses observed among patients with similar clinical and molecular profiles. Supporting our findings, the recent studies [30,51] demonstrated that models integrating quantitative radiomic features of intratumoral heterogeneity with conventional radiological assessment and clinicopathological variables significantly improves prognostic stratification and prediction of NAT response, compared with models based on radiological or radiomic features alone, supporting the biological relevance of tumor heterogeneity as a key determinant of therapeutic sensitivity. Such approaches may support safer and more effective personalization of therapy, as well as tailored surgical and follow-up strategies, with the potential to improve patient outcomes [52,53].

A key strength of our study lies in the comprehensive modeling of tumor vascularization achieved by incorporating all dynamic phases of DCE-MRI rather than restricting analysis to a single post-contrast image. By summarizing the temporal evolution of radiomic features through slope and intercept parameters, our approach captures both baseline vascular characteristics and their dynamic changes throughout contrast uptake. This contrasts with prior studies that primarily analyzed a single enhancement phase, typically assuming it best represented tumor heterogeneity [54,55,56]. Our multiphase strategy provides a more comprehensive representation of tumor physiology and may explain the improved performance observed in identifying non-responders.

This study has several limitations. First, as with any retrospective study, selection bias is an inherent issue. Second, the data were collected over an extended period where DCE-MRI protocols evolved. Although these optimizations were intended to improve image quality, they may have introduced variations that could affect the consistency of the radiomic features extracted across different time points. Third, the sample size—particularly for the non-responder subgroup—was relatively small, which may limit statistical power. Additionally, in our analyses, we specifically chose to use the third DCE-T1w images, as breast tumors were most clearly visible in this sequence. However, the modeling of the dynamics of radiomic feature was conducted using a linear regression model. While this approach is computationally efficient and simplifies the analysis of trends, it may not fully capture the complex, non-linear nature of tumor dynamics.

Future studies should aim to validate these findings in larger, prospective and multi-institutional cohorts using standardized imaging protocols. The application of advanced modeling techniques, including non-linear temporal fitting, delta-radiomics, and deep learning-based approaches, may further enhance the prediction of non-response and improve generalizability across imaging platforms.

Despite these limitations, our results support the feasibility and clinical relevance of time-dependent radiomics as a promising non-invasive imaging biomarker for identifying non-responders to neoadjuvant therapy. Incorporating temporal radiomics into the clinical workflow may facilitate earlier treatment adaptation, reduce unnecessary toxicity, and contribute to AI-guided, patient-tailored therapeutic strategies.

## 5. Conclusions

Time-dependent radiomic features from DCE-MRI can effectively predict response to NAT in breast cancer patients, with the highest accuracy in identifying non-responders, the subgroup for whom early treatment adaptation is most crucial. By capturing subtle temporal enhancement dynamics, temporal radiomics provide unique biological insights beyond static features, enabling a more precise, non-invasive assessment of therapeutic resistance. These findings highlight the potential of dynamic MRI-based AI models to support personalized, response-guided treatment strategies.

## Figures and Tables

**Table 1:** Pinder classification system describing the histopathological grading of tumor response after neoadjuvant therapy.

Pinder Classification	Explanation
1i	Pathological complete response, no DCIS
1ii	Pathological complete response, including DCIS
2i	Response > 90% (or <10% invasive tumor left)
2ii	Response 50–90% (or 10–50% invasive tumor left)
2iii	Response < 50% (or >0% invasive tumor left)
3	No signs of response

DCIS ductal carcinoma in situ. Adapted from Pinder et al. [38].

**Table 2:** Patients and tumor characteristics.

Characteristics	Center 1	Center 2
Age		
Median	51	50
IQR	46–59	43–56
Histology		
IC NST	173	39
Focality		
Unifocal	113	13
Multifocal	60	26
Grade		
G2	37	8
G3	123	29
n.a.	13	2
ER		
Positive	80	21
Negative	93	18
PR		
Positive	58	14
Negative	115	25
HER2		
Positive	102	16
Negative	71	23
Molecular subtypes		
Luminal	16	12
HER2+	99	16
Triple negative	58	11
Pre-NAT T stage		
T1	24	0
T2	111	26
T3	26	7
T4	12	6
Pre-NAT N stage		
N0	81	19
N1	91	20
N2	1	0
Pre-NAT size (mm)		
Median	30	46
IQR	22–45	27–55
Post-NAT T stage		
yT0	62	11
yTis	21	7
yT1	71	12
yT2	16	8
yT3	3	1
Post-NAT N stage		
yN0	144	29
yN1	21	4
yN2	7	5
yN3	1	1

Note: ER = estrogen receptor; PR = progesterone receptor; HER2 = human epidermal growth factor receptor 2; IC NST = Invasive Carcinoma No Special Type; IQR = interquartile range; NAT = neoadjuvant therapy; n.a.= not available.

## Data Availability

The data supporting the findings of this study are stored in an institutional database in the Excel format. Due to privacy and ethical restrictions, the data are not publicly available but may be made available from the corresponding author upon reasonable request.

## Supplementary Materials

The following supporting information can be downloaded at: https://www.mdpi.com/article/10.3390/diagnostics16040611/s1, Supplementary Material S1: Clear checklist; Supplementary Material S2: Parameters and setting for training the CNN for tumor segmentation using the third Breast DCE-MRI.; Supplementary Material S3: Parameters and settings for Radiomics features extraction.; Supplementary Material S4: Exploratory Subgroup Analysis; Supplementary Material S5: Type of response according to molecular Histotypes; Supplementary Material S6: Type of therapy; Supplementary Material S7: Confusion Matrix of Internal and External Validation Cohorts.
