# Supplementary material for: Time-Dependent DCE-MRI Radiomics to Predict Response to Neoadjuvant Therapy in Breast Cancer: A Multicenter Study with External Validation
Source: Diagnostics (Basel). 2026 Feb 19;16(4):611. doi: 10.3390/diagnostics16040611 (PMC12939715; doi:10.3390/diagnostics16040611)
Supplement: Supplementary file 1 [file diagnostics-16-00611-s001.zip › diagnostics-4144416-supplementary.pdf]

SUPPLEMENTARY MATERIAL

Supplementary material S1-Clear checklist

CLEAR Checklist v1.0

Note: Use the checklist in conjunction with the main text for clarification of all items.
Yes, details provided; No, details not provided; n/e, not essential; n/a, not applicable; Page, page number

Section	No.	Item	Yes	No	n/a	Page
**Title**						
	1	Relevant title, specifying the radiomic methodology	☑	☐	☐	
**Abstract**						
	2	Structured summary with relevant information	☑	☐	☐	
**Keywords**						
	3	Relevant keywords for radiomics	☑	☐	☐	
**Introduction**						
	4	Scientific or clinical background	☑	☐	☐	
	5	Rationale for using a radiomic approach	☑	☐	☐	
	6	Study objective(s)	☑	☐	☐	
**Method**						
Study design	7	Adherence to guidelines or checklists (e.g., CLEAR checklist)	☑	☐	☐	
	8	Ethical details (e.g., approval, consent, data protection)	☑	☐	☐	
	9	Sample size calculation	☑	☐	☐	
	10	Study nature (e.g., retrospective, prospective)	☑	☐	☐	
	11	Eligibility criteria	☑	☐	☐	
	12	Flowchart for technical pipeline	☑	☐	☐	
Data	13	Data source (e.g., private, public)	☑	☐	☐	
	14	Data overlap	☐	☑	☐	
	15	Data split methodology	☑	☐	☐	
	16	Imaging protocol (i.e., image acquisition and processing)	☑	☐	☐	
	17	Definition of non-radiomic predictor variables	☑	☐	☐	
	18	Definition of the reference standard (i.e., outcome variable)	☑	☐	☐	
Segmentation	19	Segmentation strategy	☑	☐	☐	
	20	Details of operators performing segmentation	☑	☐	☐	

Pre-processing	21	Image pre-processing details	☑	☐	☐	
	22	Resampling method and its parameters	☑	☐	☐	
	23	Discretization method and its parameters	☑	☐	☐	
	24	Image types (e.g., original, filtered, transformed)	☑	☐	☐	
Feature extraction	25	Feature extraction method	☑	☐	☐	
	26	Feature classes	☑	☐	☐	
	27	Number of features	☑	☐	☐	
	28	Default configuration statement for remaining parameters	☑	☐	☐	
Data preparation	29	Handling of missing data	☐	☑	☐	
	30	Details of class imbalance	☑	☐	☐	
	31	Details of segmentation reliability analysis	☑	☐	☐	
	32	Feature scaling details (e.g., normalization, standardization)	☑	☐	☐	
	33	Dimension reduction details	☑	☐	☐	
Modeling	34	Algorithm details	☑	☐	☐	
	35	Training and tuning details	☑	☐	☐	
	36	Handling of confounders	☑	☐	☐	
	37	Model selection strategy	☑	☐	☐	
Evaluation	38	Testing technique (e.g., internal, external)	☑	☐	☐	
	39	Performance metrics and rationale for choosing	☑	☐	☐	
	40	Uncertainty evaluation and measures (e.g., confidence intervals)	☑	☐	☐	
	41	Statistical performance comparison (e.g., DeLong's test)	☑	☐	☐	
	42	Comparison with non-radiomic and combined methods	☑	☐	☐	
	43	Interpretability and explainability methods	☐	☐	☐	
**Results**						
	44	Baseline demographic and clinical characteristics	☑	☐	☐	
	45	Flowchart for eligibility criteria	☑	☐	☐	
	46	Feature statistics (e.g., reproducibility, feature selection)	☑	☐	☐	
	47	Model performance evaluation	☑	☐	☐	
	48	Comparison with non-radiomic and combined approaches	☑	☐	☐	
**Discussion**						

	49	Overview of important findings	☑	☐	☐	
	50	Previous works with differences from the current study	☑	☐	☐	
	51	Practical implications	☑	☐	☐	
	52	Strengths and limitations (e.g., bias and generalizability issues)	☑	☐	☐	
**Open Science**						
Data availability	53	Sharing images along with segmentation data [n/e]	☐	☑	☐	
	54	Sharing radiomic feature data	☐	☑	☐	
Code availability	55	Sharing pre-processing scripts or settings	☑	☐	☐	
	56	Sharing source code for modeling	☑	☐	☐	
Model availability	57	Sharing final model files	☐	☑	☐	
	58	Sharing a ready-to-use system [n/e]	☐	☑	☐	

Kocak B, Baessler B, Bakas S, Cuocolo R, Fedorov A, Maier-Hein L, Mercaldo N, Müller H, Orlhac F, Pinto Dos Santos D, Stanzione A, Ugga L, Zwanenburg A. CheckList for EvaluAtion of Radiomics research (CLEAR): a step-by-step reporting guideline for authors and reviewers endorsed by ESR and EuSoMII. Insights Imaging. 2023 May 4;14(1):75. doi: 10.1186/s13244-023-01415-8

Supplementary Material S2- Parameters and setting for training the CNN for tumor segmentation using the third Breast DCE-MRI.

**Parameter**	**Setting**
Convolution Kernel	3 Dimensional
Batch size	2
Intensity Normalization Algorithm	Z-Score
Voxel Spacing	0.722 mm x 0.722 mm x 2.0
Data Augmentation	Spatial (Rotation, Translation, Resize, Resample) and Intensity (Brightness, Contrast, Blurring, Sharpening).

Supplementary Material S3: Parameters and settings for Radiomics features extraction. Tumor VOI segmentations have been extracted from each DCE MRI sequence.

**Parameter**	**Setting**
Bin width	50
Normalize	True
Normalize scale	100
Voxel Array Shift	300
Interpolation	Linear
Resample Pixel Spacing	1x1x1 mm

Supplementary material S4- Exploratory Subgroup Analysis

To further investigate the temporal dynamics of radiomic features, an exploratory subgroup analysis was conducted by reclassifying patients into three categories according to the Pinder classification: pathologic complete response (pCR), defined as complete pathological response with no residual invasive or in situ carcinoma (Pinder 1i); pathologic complete response in situ (pCRis), corresponding to complete response of the invasive component with persistence of ductal carcinoma in situ (Pinder 1ii); and partial or non-response (pPR/pNR), including Pinder 2i, 2ii, 2iii, and 3. This refined classification allowed a more accurate separation of true complete responders and made it possible to evaluate whether residual in situ disease exhibited enhancement kinetics more similar to complete response or to residual invasive disease. Combining partial and non-responding cases avoided excessively small subgroups and ensured more robust statistical comparisons.

In this refined subgroup analysis, dynamic radiomic features extracted from DCE-MRI were compared across the three response categories. Among all the parameters analyzed, the slope of the wavelet-GLCM LHH Imc2 and the intercept of first-order Kurtosis showed statistically significant differences among groups ($p < 0.05$). The wavelet-GLCM LHH Imc2 reflects the degree of heterogeneity within the voxel intensity texture, whereas kurtosis quantifies deviation from a normal intensity distribution, describing how peaked or flat the signal histogram appears.

Regarding the slope of wavelet-GLCM LHH Imc2, pCR patients showed a more concentrated and peaked signal at maximum enhancement, suggesting greater regularity in texture evolution, while pCRis and pPR/pNR patients exhibited flatter and more variable signal distributions over time, consistent with a more heterogeneous enhancement pattern. Similarly, the intercept of first-order kurtosis revealed that pCR lesions maintained relatively stable values across post-contrast phases, reflecting a homogeneous internal composition, whereas pCRis and pPR/pNR lesions displayed progressively flatter distributions as the dynamic series evolved, indicating increasing intratumoral heterogeneity.

The final radiomics model trained on these refined subgroups achieved an overall accuracy of 73.8% (31/42), with a macro-averaged sensitivity of 71.8% and a specificity of 73.6% in the internal validation cohort. In the external validation cohort, the model reached an accuracy of 71.7% (28/39), with a macro-averaged sensitivity of 75.2% and a specificity of 68.8%. The corresponding AUC values were 0.89 for pCR versus the

rest, 0.88 for pCRis versus the rest, and 0.85 for pPR/pNR versus the rest in the internal validation, and 0.80, 0.71, and 0.75, respectively, in the external validation cohort.

Figure S1

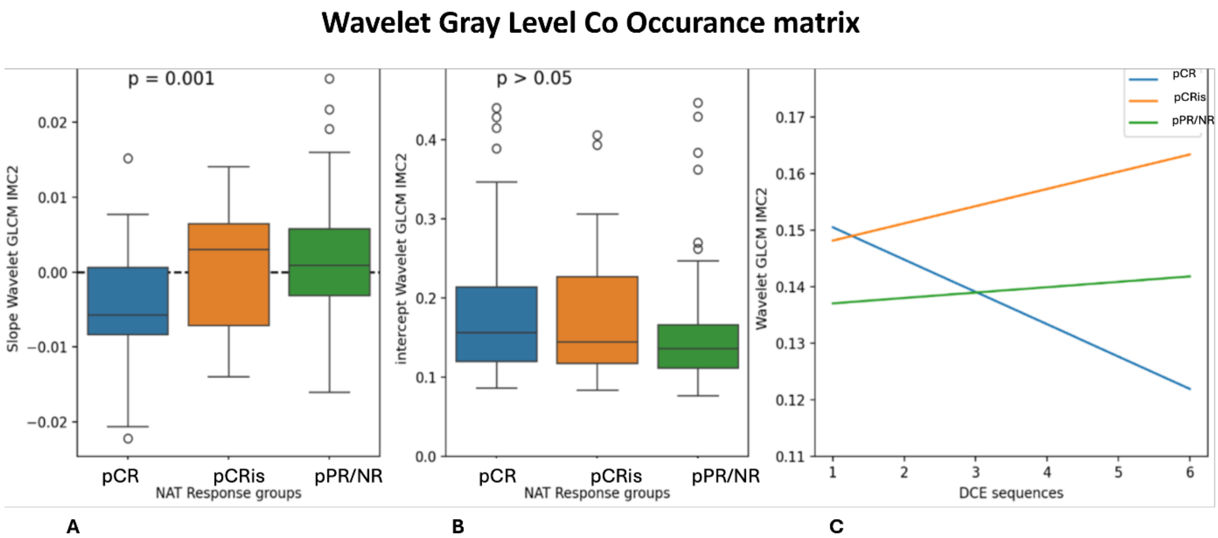

Boxplot and Lineplot showing the dynamic changes of the GLCM Slope Wavelet GCLCM. A: slope; B: intercept; C: average linear regression model of each NAT response groups

Figure S2

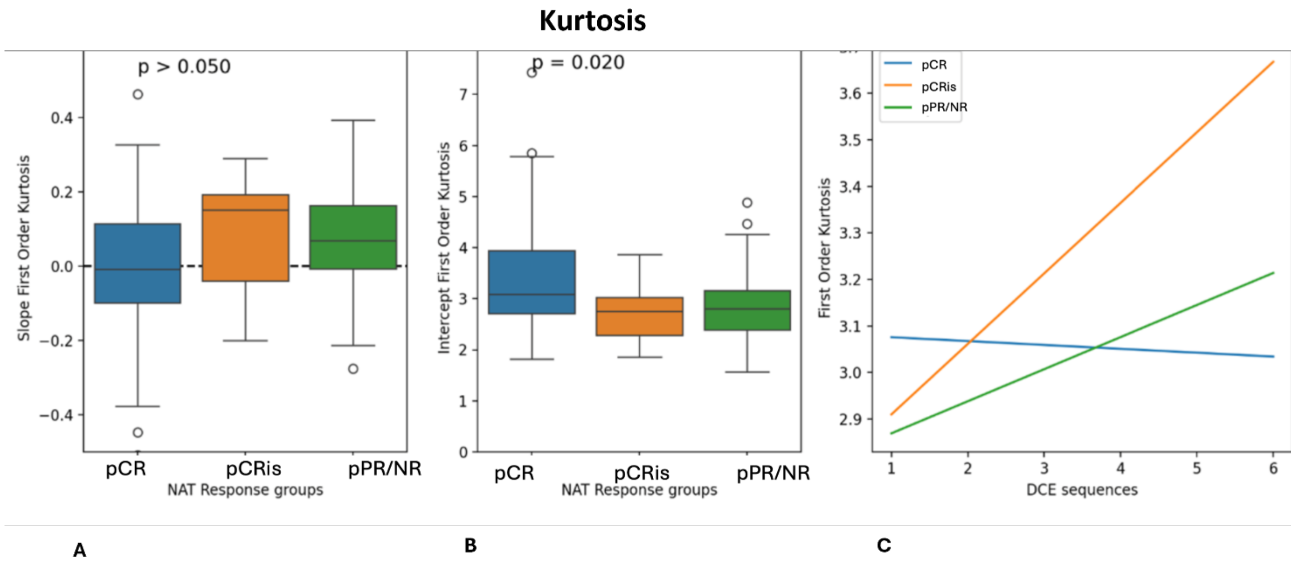

Boxplot and Lineplot showing the dynamic changes of the Slope first order kurtosis. A: slope; B: intercept; C: average linear regression model of each NAT response groups

Supplementary material S5- Type of response according to molecular Histotypes

Center 1					
	CR	CR is	PR	NR	TOT
Luminal A	2	0	2	0	4
Luminal B	5	0	7	0	12
Her 2+	33	17	47	2	99
TN	22	4	30	2	58
TOT	62	21	86	4	173
**Center 2**					
	CR	CR is	PR	NR	TOT
Luminal A	0	0	0	0	0
Luminal B	4	1	6	1	12
Her 2+	3	5	8	0	16
TN	6	1	3	1	11
TOT	13	7	17	2	39

CR: complete response (no DCIS); CR is: complete response (DCIS associated); PR : partial response; NR: Non response. TN: triple negatives.

Supplementary material S6- Type of therapy

**Type of Therapy**	**Center 1**	**Center 2**
Anti-HER2 therapies Trastuzumab ± Pertuzumab combined with Taxanes ± AC	101	16
Immunotherapy with Pembrolizumab associated to Taxanes ± AC	2	10
Conventional chemotherapy (non-targeted) (AC± Taxanes)	54	11
Chemotherapy with Carboplatin (without immunotherapy)	15	0
Hormonal therapy (Letrozole + CDK inhibitor)	1	2

AC: Anthracycline-based regimen (Doxorubicin + Cyclophosphamide)

Supplementary material S7: Confusion Matrix of Internal (25% total Cohort 1) and External Validation Cohorts

		Internal Validation Cohort		
		**Predicted**		
		pCR	pPR	pNR
**True**	**pCR**	12	7	1
	**pPR**	5	16	0
	**pNR**	0	1	1
		**External Validation Cohort**		
		**Predicted**		
		pCR	pPR	pNR
**True**	**pCR**	16	4	0
	**pPR**	5	11	1
	**pNR**	1	0	1
